# Distribution of sugar-sweetened beverage sales volume by sugar content in the United States: implications for tiered taxation and tax revenue

**DOI:** 10.1057/s41271-019-00217-x

**Published:** 2020-01-22

**Authors:** Lisa M. Powell, Tatiana Andreyeva, Zeynep Isgor

**Affiliations:** 1 Division of Health Policy and Administration, School of Public Health, University of Illinois at Chicago, Chicago, IL USA; 2 Department of Agricultural and Resource Economics, University of Connecticut, Storrs, CT USA

**Keywords:** Sugar-sweetened beverages, Sweetened beverage taxes, Sugar content, Tax policy, Fiscal policy

## Abstract

This study draws on data on sales volume, brand-level market shares, and sugar content to calculate the distribution of sugar-sweetened beverage (SSB) sales volume by sugar content, propose sugar content thresholds for a tiered tax structure, and estimate tax revenue. The most common SSBs sold had 26 g of sugar/8-oz serving; 70.8% had ≥ 25 g of sugar/8-oz serving, 16.9% were in the 10–15 g range, and 8.7% were in the 16–20 g range. A tiered tax with cut points at < 20 g and < 5 g of sugar/8-oz serving is proposed. A tax of 1¢/oz for SSBs in the second tier and 2¢/oz in third tier is projected to raise $18.2 billion in tax revenue similar to the 1.5¢/oz flat tax projection ($18.0 billion) but would yield 9% lower SSB volume. Understanding the distribution of SSB sales volume by sugar content informs policymakers on tiered tax structures, which may discourage consumption of SSBs with high levels of sugar and incentivize reformulation.

## Introduction

In 2016, 1.9 billion or 39% of adults and 381 million children and adolescents worldwide were overweight or obese [1]. The global prevalence of diabetes in 2014 was 9% [2]. Sugar-sweetened beverages (SSBs) (beverages with added caloric sweeteners) can be a significant source of discretionary calories globally and, for example, provide almost half of all added sugars consumed in the diet of those living in the United States (USA) [3, 4]. The negative health effects of SSB consumption including weight gain and increased risk of type 2 diabetes, cardiovascular disease, dental caries, and osteoporosis [5, 6]—along with related economic costs [7, 8]—have led to international recommendations for governments to use fiscal policy measures to reduce consumption and improve health [4]. More than 40 countries worldwide have implemented SSB taxes [9].

Beverage taxes to date have mostly used a ‘flat tax’ per unit volume. This means that taxed beverage products are subject to the same tax irrespective of their sugar content. While the volume-based flat tax has the important advantage of simplicity in implementation, it does not provide incentives for consumers to switch to less calorically sweetened beverages or for the beverage industry to reformulate products to reduce sugar content per serving. Evaluations of Mexico’s flat tax of 1 peso/L on SSBs found a decline in SSB sales sustained 2 years post-tax [10, 11] and a recent evaluation of the 10% flat tax in Barbados also found a significant reduction in SSB sales [12]. Recent evaluations of local US flat excise sweetened beverage taxes ranging from one to two cents per ounce also showed reductions in SSB sales/purchases post-tax [13–17].

A limited number of countries have proposed and implemented a tiered tax approach, taxing beverages at different amounts depending on their sugar content (e.g., grams (g) of sugar per unit of volume or serving). In 2014, Chile effectively created a tiered tax by increasing their SSB tax rate from 13 to 18% on SSBs with high levels of sugar (H-SSBs: > 6.25 g sugar/100 ml) and reducing it from 13 to 10% on SSBs with no or low levels of sugar (L-SSBs: < 6.25 g sugar/100 ml, including zero-calorie artificially sweetened beverages). Recent evaluations showed a reduction in purchases of H-SSBs with no change or an increase in L-SSB purchases [18, 19]. In April 2018, the United Kingdom (UK) implemented a three-tiered soft drink industry levy (SDIL) with no tax on beverages with < 5 g of sugar/100 ml, and 18 pence (p)/L and 24p/L on beverages with 5–8 g and > 8 g of sugar/100 ml, respectively. As a result of industry reformulation in reaction to the SDIL announcement, within two years, there was an 11% reduction in sugar content of drinks subject to the levy, and the caloric content of such drinks fell by 6% [20]. Also in April 2018, South Africa introduced a SSB tax, the Health Promotion Levy (HPL), based on a continuum (rather than discrete tiers) of sugar content in SSBs at a rate of 0.021 ZAR (~ 0.15 US cents) per gram of sugar (over an exempt amount of 4 g/100 ml). A recent study found that in the first year following the introduction of the HPL, taxed beverage prices increased similarly for low- and high-sugar beverages and industry reformulated its products [21].

The tiered tax approach has drawn growing interest globally, but questions remain about the appropriate tax tier thresholds in terms of impacts on consumption, reformulation, and tax revenue. To our knowledge for countries in which tiered tax structures are in place, there exists no documented analysis on how the tax tier thresholds were developed. Understanding the actual distribution of the most commonly consumed SSBs by sugar content will help guide the choice of meaningful tax tier thresholds. This study assesses the distribution of SSB sales volume in the USA by beverage sugar content, including by beverage category and socio-demographic SSB consumption patterns, and proposes a potential tiered tax structure. Based on the observed distribution of SSB volume by sugar content, we provide revenue estimates for a proposed three-tiered tax and compare them to flat tax revenue projections. This study aims to inform policymakers about potential thresholds for a SSB tiered tax structure in the USA and its revenue generation implications.

## Methods

### Distribution of SSB sales volume

We made use of data from the Beverage Marketing Corporation (BMC) and the UConn Rudd Center Sugary Drink Tax Calculator (UConn Calculator) 2018 projections [22]. The BMC data included brand-level information on 2016 US total SSB sales volume, market shares, and sugar content in grams per 8-ounce (oz) serving for each SSB category. These included the following SSB categories: carbonated soft drinks (CSDs) or sodas, fruit drinks, sports drinks, value-added (enhanced) water, ready-to-drink (RTD) tea and coffee, and energy drinks. We did not include fruit drink powder mixes, diet (zero-calorie) products, other non-SSB products such as 100% fruit juices. Individually listed brands included products with added caloric sweeteners and, across the different beverage categories, accounted for 78–98% of the total volume sold in 2016 in the USA. We assumed that brands with very small market shares would have a similar sugar content distribution as the average of brands within each SSB category. To assure data reliability, we cross-checked the BMC 2016 data with other sources; for example, the match with 2016 data in Beverage Digest was 98.7% for CSD totals and within 95–99% for major CSD brands [23].

Using the brand-level sales volume shares from the BMC 2016 data, we allocated 2018 volume projections from the tax calculator across different brands for each SSB category. We then summed up total volume for the brands by sugar content per 8-oz within each category and across all SSBs.

We assessed the extent to which SSB consumption by socio-demographic segments (by sex, age, race/ethnicity, and education) of the population would fall into the proposed tax tiers based on the sugar content of the categories and amounts of SSBs they currently consume. The 2018 total volume projection allocated to each brand within each SSB category was further allocated into each of the socio-demographic categories based on the US population shares from the American Community Survey (ACS) 2016 1-year data file of the US Census Bureau [24]. We adjusted these by the consumption shares of each SSB category based on the National Health and Nutrition Examination Survey (NHANES) 2013–2014 24-h Day 1 dietary recall data [25]. Finally, we generated total volume for each socio-demographic attribute by grams of sugar per 8-oz serving across all SSBs and obtained per capita volume sold based on their respective US population totals.

### Tiered tax revenue

We estimated tiered tax revenue for the USA and by state. We derived the state-level sales data from regional sales data projected to 2018 from the UConn Calculator, that were adjusted for each state’s SSB consumption patterns based on its socio-demographic composition (sex, age, race/ethnicity, and education) using NHANES 2013–2014 and US Census data. Detailed methods for estimating national and state-level beverage sales are available elsewhere, including assumptions about the price elasticity (− 1.21), full (100%) tax pass-through, constant prices across states, and full compliance with tax payments [22, 26, 27]. Using the brand-level total sales volume shares in the BMC 2016 data, we allocated 2018 volume projections for the USA as a whole and for each state from the UConn Calculator across different brands listed within each SSB category. We then summed up total volume for the brands by sugar content within each SSB category and across all SSBs.

We present SSB tax revenue estimates that compare scenarios for flat tax rates of $0.01/oz, $0.015/oz, and $0.02/oz with a tiered tax structure of no tax for the first tier, $0.01/oz for the second tier, and $0.02/oz for the third tier.

## Results

### Distribution

**Fig. 1:**
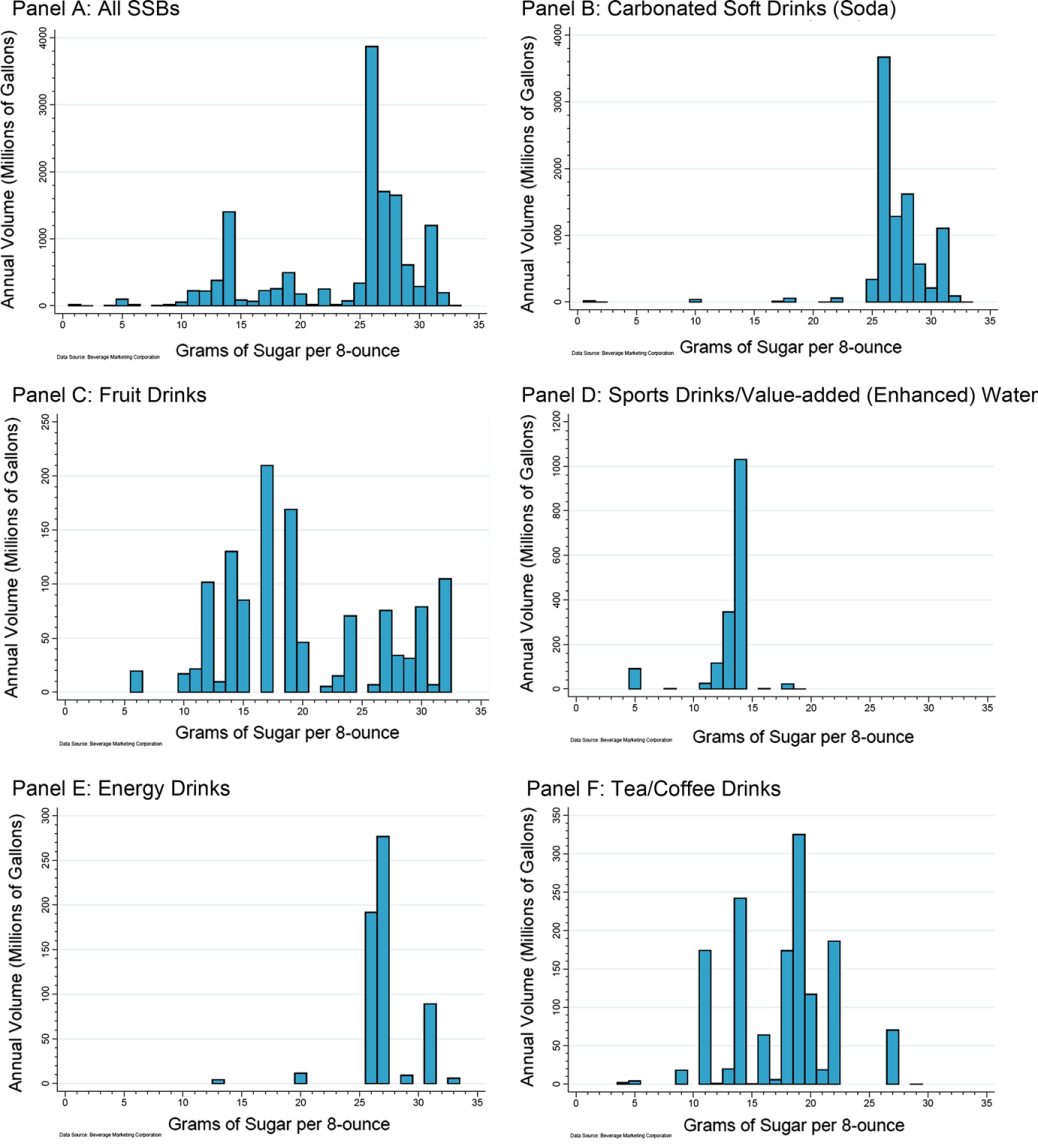
Distribution of annual sugar-sweetened beverage (SSB) sales volume by sugar content, all SSBs and by SSB category, US total, 2018

The analysis of the overall US SSB market for 2018 revealed a number of ‘spikes’ in the sales volume distribution of beverages by sugar content per 8-oz serving. Panel A in Fig. 1 shows that by far the largest single portion of SSBs sold were for those with 26 g of sugar/8-oz serving (almost 4 billion gallons; 11.8 gallons per capita), such as sodas and energy drinks. A distant second and third at just over 1.5 billion gallons each were beverages with 28 g and 27 g of sugar/8-oz serving, again made up mainly from sodas and energy drinks. Overall, 70.8% of SSB sales were for products in the high-sugar range of ≥ 25 g of sugar/8-oz, with 66.9% of SSB sales in range of 26–31 g of sugar/8-oz. Next, there were two clusters of sales volume by sugar content observed in the middle part of the distribution: one cluster with a peak at 14 g of sugar/8-oz serving (16.9% in the 10–15 g range) that included mostly sports drinks and value-added (enhanced) water, fruit drinks, and RTD teas/coffees. Another smaller cluster occurred around 19 g of sugar/8-oz (8.7% in the 16–20 g range) including mostly fruit drinks and RTD teas/coffees. Volumes for calorically sweetened beverages with < 10 g of sugar/8-oz were very low (1.1%).

There were considerable differences in the volume distribution by sugar content across beverage categories (shown in Panels B through F in Fig. 1). Almost the entire volume of sodas (98.1%) and energy drinks (97.3%) was for high-sugar products with ≥ 25 g of sugar/8-oz. For fruit drinks, 60.6% of sales were in the range of 12–20 g of sugar/8-oz and 27.3% in the high-sugar range. The volume of RTD teas/coffees spanned across the sugar content spectrum and sports drinks and value-added waters was mostly clustered at 12–14 g of sugar/8-oz (91.3%).

### SSB tiered tax structure

The sugar content analysis revealed a dominant cluster of beverage sales in the high range of ≥ 25 g of sugar/8-oz. Next, we observed a cluster in the mid-range of 10–15 g, with an adjacent cluster from 16 to 20 g of sugar/8-oz. The identification of these clusters across the sugar content spectrum constitutes a population data-based approach for the development of thresholds for sugar content in a tiered tax structure for SSB taxes. To discourage consumption of high-sugar beverages and incentivize industry reformulation, sugar content thresholds for differential tax amounts per unit of volume should be implemented at meaningful distances below the major cluster ranges. In this regard, a public health action plan that was foundational to the development of the SSB tiered tax in the UK set a goal to reduce sugar levels in foods and beverages by 20% [20]. This would suggest a reduction in sugar for the cluster of high-range (≥ 25 g of sugar/8-oz) sugar content beverages by a minimum of 5 g to a threshold of under 20 g of sugar/8-oz. Using this same level of reduction suggests a threshold of under 5 g of sugar/8-oz for the lower bracket. This would place it at equal distance from the 10 g lower bound of the mid-range cluster, although there is a larger percentage change in relation to the mid-range cluster. Using the 5 g minimum reduction as a basis for a meaningful reduction in sugar content, the pattern of the SSB volume distribution shown in Panel A in Fig. 1 reveals that thresholds for sugar content at 20 g per 8-oz (equivalent to 8.4 g per 100 ml) and 5 g per 8-oz (equivalent to 2.1 g per 100 ml) may reduce consumption and incentivize reformulation. Thus, based on the observed volume distribution by sugar content, we recommend the following three-tiered tax structure:

Tier 1 (T1): No tax for SSBs with sugar content of > 0 g and < 5 g per 8-oz
Tier 2 (T2): Lower tax rate for SSBs with sugar content of ≥ 5 g and < 20 g per 8-oz
Tier 3 (T3): Higher tax rate for SSBs with sugar content of ≥ 20 g per 8-oz

Based on the distribution of SSB volume by sugar content, only 0.1% of volume sold currently would fall into T1 and not be taxed, 25.3% would fall into T2, and 74.6% would fall into the highest tax tier of T3. For the overwhelming majority of SSBs, that contain high amounts of sugar at ≥ 25 g of sugar/8-oz, reformulation that reduces added sugar in these beverages to under 20 g/8-oz would represent a 20% to 38% reduction in sugar content.

**Fig. 2:**
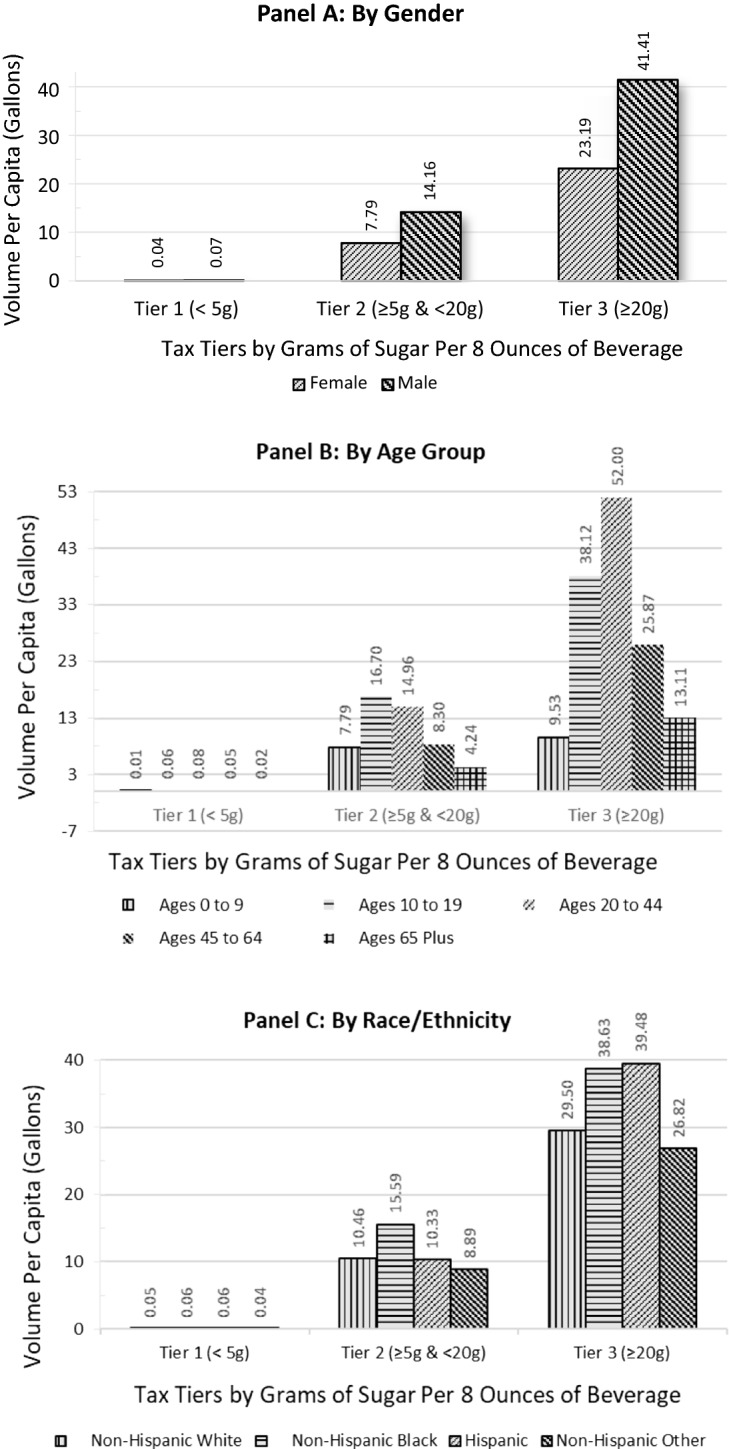

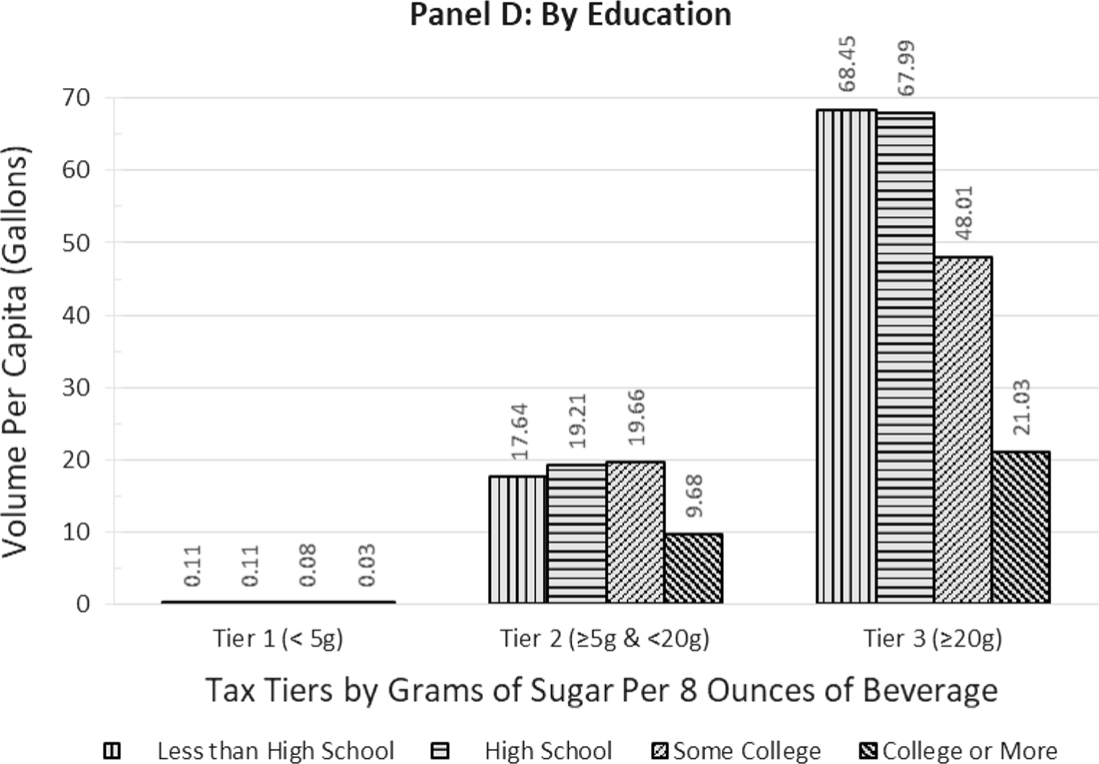
Per capita 2018 sales volume of sugar-sweetened beverages by tax tiers, by gender (**a**), age group (**b**), race/ethnicity (**c**), and education (**d**)

Panels A through D in Fig. 2 depict the SSB volume distribution across the tax tiers by gender, age, race/ethnicity, and education. Panel A shows that males account for more of the volume in both T2 and T3 compared to females, but similarly for both genders, roughly three times the SSB volume falls into T3 versus T2. Panel B shows considerable variation across the tiers by age. Young adults (20–44-year-olds) consume the highest per capita volume of T3 beverages whereas youths aged 10–19 consume the highest per capita volume of T2 SSBs, including a substantial portion of sports drinks. Further, 2.3 to 3.5 times the SSB volume per capita falls into T3 versus T2 for all age groups, except children aged 0–9 who have almost similar volumes in T3 and T2. By race/ethnicity, Panel C shows that non-Hispanic black and Hispanic persons have the highest absolute per capita volumes in T3, and Hispanics have the highest relative consumption of 3.8 times the volume in T3 versus T2. Non-Hispanic black persons also consume the highest absolute volume of beverages in T2. By education (for adults only), Panel D shows that lower-educated adults with no college consume the highest per capita volume of SSBs in T3 while also having the highest proportion of volume in T3 versus T2; a ratio of 3.9 and 3.5 for those with less than high school-level education and high school diploma, respectively, compared to 2.4 and 2.2 times for their counterparts with some college and college degree or more, respectively.

### Tax revenue

Table 1 shows that increasingly higher flat tax amounts per SSB ounce of 1¢/oz, 1.5¢/oz, and 2¢/oz are estimated to raise $13.9 billion, $18.0 billion, and $20.1 billion dollars in tax revenue, respectively. Note that as the flat tax rate doubles, for example, from 1¢/oz to 2¢/oz, tax revenue less than doubles due to the predicted decline in consumer demand that is assumed to be elastic. A flat tax of 2¢/oz versus 1¢/oz raises 44% more tax revenue and results in lower net SSB volume (7.9 versus 10.9 billion gallons, respectively; data not shown). Table 1 also provides flat tax and tiered tax revenue estimates by state.

**Table 1:** Sugar-sweetened beverage (SSB) tax revenue for the USA and by state, by flat tax and tiered tax structures, 2018

	Flat tax structure			Tiered tax structure
	$0.01¢/oz	$0.015¢/oz	$0.02¢/oz	T1: $0.00¢/oz T2: $0.01¢/oz T2: $0.02¢/oz
	2018 millions of dollars per year			
USA	13,949.4	18,004.6	20,113.4	18,168.5
By state				
Texas	1214.7	1562.5	1737.2	1568.5
California	1145.3	1510.2	1736.6	1513.7
Florida	956.3	1230.0	1367.4	1235.8
New York	830.5	1071.5	1196.2	1074.0
Illinois	612.0	786.4	873.0	800.3
Ohio	573.5	736.7	817.5	750.4
Pennsylvania	549.0	708.0	789.9	710.3
Michigan	483.9	621.7	690.1	632.8
Georgia	476.5	612.9	681.4	615.4
North Carolina	463.5	596.3	663.1	598.6
New Jersey	370.8	478.4	534.1	479.3
Virginia	367.5	472.8	525.8	474.1
Missouri	350.4	448.4	494.9	460.9
Indiana	333.7	428.6	475.5	436.8
Arizona	304.3	394.4	443.1	405.8
Tennessee	309.5	398.0	442.4	400.0
Minnesota	303.4	388.4	428.9	398.8
Wisconsin	281.2	361.2	400.9	367.8
Massachusetts	276.4	356.6	398.2	357.0
Maryland	250.8	323.7	361.5	323.9
Kentucky	234.3	301.1	334.6	303.7
South Carolina	231.2	297.4	330.7	298.7
Alabama	226.2	290.9	323.4	292.3
Louisiana	222.2	285.8	317.6	287.3
Colorado	211.1	275.1	311.2	279.9
Washington	208.9	275.5	316.8	275.5
Iowa	179.3	229.4	253.2	235.8
Oklahoma	168.3	216.5	240.7	217.2
Kansas	162.8	208.3	230.0	214.0
Connecticut	145.9	188.3	210.2	188.6
Mississippi	141.1	181.5	201.8	182.4
Arkansas	140.6	180.8	201.0	181.8
Nevada	121.6	158.4	179.1	161.7
Utah	120.4	156.8	177.5	159.7
Oregon	117.8	155.3	178.6	155.4
Nebraska	107.9	138.1	152.5	141.9
West Virginia	97.6	125.4	139.2	126.6
New Mexico	90.9	117.8	132.3	121.3
Idaho	67.3	87.6	99.2	89.4
Maine	55.7	71.9	80.2	72.1
New Hampshire	54.5	70.3	78.5	70.4
South Dakota	50.0	63.9	70.6	65.7
North Dakota	44.4	56.8	62.7	58.3
Rhode Island	44.3	57.1	63.7	57.2
Hawaii	41.1	54.2	62.3	54.3
Montana	40.6	52.9	59.9	53.9
Delaware	41.4	53.4	59.6	53.5
Vermont	25.4	32.7	36.5	32.8
Wyoming	23.2	30.2	34.2	30.8
Alaska	21.7	28.6	32.9	28.7

T1: Tier 1 SSBs with sugar content of > 0 g and < 5 g per 8-oz. T2: Tier 2 SSBs with sugar content of ≥ 5 g and < 20 g per 8-oz. T3: Tier 3 SSBs with sugar content of ≥ 20 g per 8-oz. States are listed in order by amount of tax revenue raised under the tiered tax structure

A tiered tax of 1¢/oz for T2 SSBs with sugar content of ≥ 5 g and < 20 g per 8-oz and 2¢/oz for T3 SSBs with sugar content of ≥ 20 g per 8-oz is projected to raise $18.2 billion in total SSB tax revenue across the two tiers, which is just slightly higher than the tax revenue projection of $18.0 billion for a flat tax amount of 1.5¢/oz (the mean of the two tiered amounts) on all SSBs. The tiered tax is projected to lead to 9% lower SSB volume as compared to the 1.5¢/oz flat tax (8.5 versus 9.4 billion gallons, respectively) due to a higher tax on a greater proportion of the overall volume in T3 and a related larger reduction in SSB volume.

In sensitivity analyses, we re-estimated tax revenue for the tiered tax to account for potential reformulation in each beverage category, keeping all other model assumptions the same. Given that reformulation led to an 11% reduction in sugar content in beverages following the introduction of the SDIL in the UK, we first considered reformulation where 10% of volume in T3 moves into T2 and 10% of volume in T2 moves into T1. Next, because the UK’s top tier tax rate is slightly less than one half of the 2-cent/oz top tier rate in this study, we considered a second scenario where 20% of volume in T3 moves into T2 and 20% of volume in T2 moves into T1. Overall, tax revenue from the tired tax fell from $18.2 billion with no reformulation to $17.4 billion, a 4.3% reduction in revenue, under the assumption of 10% reformulation, to $16.6 billion, a 8.9% reduction in revenue, under the assumption of 20% reformulation (data not shown in tables). As expected, under both reformulation scenarios, volume was lower in T3 and higher in T2 and T1. It is higher in T2 because the shift in volume from T3 to T2 was greater than the shift from T2 to T1 (since the greatest volume originated in T3). Thus, although revenue is lower with reformulation, so too is the volume of beverages falling into the highest sugar content tier: under the tiered tax (compared to no tax) beverage volume in T3 falls by 45.4% assuming no reformulation, by 50.9% under the 10% reformulation assumption, and by 56.4% under the 20% reformulation assumption.

## Discussion

This study assessed the distribution of SSB volume sold along the sugar content spectrum for the USA, including differences by beverage category and population groups. The analysis revealed multiple clusters of SSB volume by sugar content, particularly in the ranges of 26–31 g and 11–14 g of sugar/8-oz. The observance of these clusters serves as a basis for the development of thresholds for sugar content in a tiered tax approach for SSB taxes. In order to discourage consumption of high-sugar beverages and incentivize meaningful beverage reformulation, sugar content thresholds for different tiers and tax rates could be implemented at a distance of approximately 5 g below the lower bound of the dominant cluster areas—with cut points for tax rates at < 20 g and < 5 g of sugar/8-oz.

Given that about seven in ten SSBs contain 25 g or more of sugar/8-oz, moving consumers from the highest tax bracket to the next one would represent a 20–38% reduction in sugar content of those consumed beverages. This could be achieved through reformulation that ‘moves’ products out of the high tax bracket or through behavior change that reduces consumption of SSBs with high levels of sugar via substitution to beverages with under 20 g of sugar/8-oz or both. Assessing sugar content by SSB category showed that sodas and energy drinks predominantly fall into high-sugar T3. Further, young adults and youths, Hispanics, and lower-educated adults are relatively heavier consumers of the high-sugar T3 SSBs relative to SSBs with lower sugar content. Thus, the higher T3 tax amount will impact the products that they consume the most and, in turn, would be expected to particularly reduce their sugar intake from such SSBs.

The extent to which and how beverage companies respond to a tiered tax structure with reformulation remains an open question. It is unlikely that reformulation will occur in response to local taxes implemented in small markets. Faced with SSB taxes in larger (national) markets producers could reformulate by reducing the sugar content or by replacing the caloric sweeteners with artificial sweeteners to maintain the same level of sweetness in drinks for consumers. The tiered tax thresholds in the UK (equivalent to 11.8 g/8-oz and 18.9 g/8-oz) are similar to our highest tier of 20 g/8-oz but allows for substantially more sugar content in beverages that are not taxed (11.8 g vs. 5 g/8-oz). Based on the impending imposition of the SDIL at the national level, industry in the UK responded with an 11% reduction in sugar levels per 100 ml of drinks subject to the levy prior to its implementation. Chile’s threshold for its two tax rates is equivalent to 14.8 g/8-oz where all sweetened beverages are subject to a minimum of the lower tax rate; that is, there is no bottom tax-free tier, including for zero-calorie artificially sweetened diet beverages. Recent evidence from the South African HPL imposed on the linear continuum of sugar content in SSBs found heterogeneity in reformulation with some brands substantially reducing sugar content (from more than 10 g per ml to under 5 g per ml) despite the fact that there are no specific target thresholds below which the tax rates fall [21]. Continued evaluation of reformulation across all types of sugar content-based tax designs is important to understand which designs may be most effective in bringing down overall sugar levels.

Without any reformulation and assuming no tax avoidance, based on the distribution of sugar content in SSBs currently sold in the USA, we estimated that a national tiered tax of 0¢/oz for T1, 1¢/oz for T2, and 2¢/oz for T3 SSBs would raise $18.2 billion in total tax revenue, which is just slightly higher than the tax revenue projection of $18.0 billion for a flat tax amount equal to the average of the T2 and T3 tiered amounts at 1.5¢/oz. However, because the 70% of SSBs fall into T3 at the higher tax amount, we estimated that a tiered tax structure with no reformulation would to lead to 9% lower volume of SSBs nationally compared to the flat tax. Overall, relative reductions in sugar would be even greater under the tiered tax structure since the greater volume reduction corresponds to the high-sugar-content beverages. Finally, we noted that with reformulation, revenue under the tiered tax would be expected to be lower as more beverages become subject to the lower T2 tax rate and to no tax for T1. Indeed, in two examples, we showed that if either 10% or 20% of products moved from T3 to T2 and from T2 to T1, revenue raised under the tiered tax would fall from $18.2 billion with no reformulation to $17.4 billion under the 10% reformulation assumption, to $16.6 billion with 20% reformulation.

This study is the first to our knowledge to assess the distribution of a country’s sales volume of SSBs by sugar content to inform on outcomes for a tiered tax structure. It is subject to several limitations and assumptions. Foremost, in order to calculate revenue estimates, we needed to make a number of assumptions. First, we assumed an average price elasticity (− 1.21) from the literature that may differ across beverage categories and populations and, hence, tiers. Second, our assumption of full compliance with the tax implementation and no tax avoidance is likely optimistic; this issue, however, is applicable for any SSB tax, flat or tiered. Therefore, all revenue estimates should be considered as upper bounds. Third, we also assumed that the taxes for the flat tax and for each tier were fully passed on to consumers, yet evidence from the USA reveals a range of tax pass-through from partial to over-shifting with heterogeneity by store type and beverage categories and sizes [28]. Further, no evidence exists on tax pass-through for discrete tiered taxes and one recent study on the South African HPL based on sugar content per gram showed that prices rose similarly for high- and low-sugar beverage products rather than linearly based on the tax design [21]. Another limitation for assessing revenue is that the exact extent of reformulation is unknown and will differ based on a number of factors, such as the size of the tax (the size of the incentive) and the size of the market (the population in the tax jurisdiction). Indeed, research into determinants and extent of industry reformulation is needed.

Understanding the distribution of SSB sales volume by sugar content is important to inform policymakers about appropriate thresholds in a tiered tax structure for reducing consumption and incentivizing reformulation. Indeed, the finding from this study is an example that applies to the USA and any jurisdiction considering a tiered tax should undertake a similar exercise to determine appropriate tax tier thresholds. Further, observed clusters will inform on thresholds and the number of thresholds, but policymakers in the taxing jurisdictions will still need to determine how far down from the clusters they place the thresholds and what differential tax rates to apply at each threshold. Both of these factors should be tied to goals for sugar content reduction as they will differentially incentivize both reformulation and behavior change. Further, if the observed distribution appears to be more continuous without a discrete set of clusters, this may suggest alternate sugar content-based models such as a linear SSB tax per gram of sugar (i.e., the South Africa HPL). While taxes based on sugar content may offer added incentives for product reformulation and greater incentives for behavior change for the most sugary drinks, they may not be appropriate in jurisdictions that do not have strong tax administration [4]. Thus, when assessing SSB tax designs based on either discrete or continuous levels of sugar content compared to flat volume-based taxes, it is also important to do so in the context of tax administration capacity. Finally, as countries or local jurisdictions within countries implement SSB tax structures based on sugar content, it will be critical to evaluate the net effect on sugar intake resulting from both reformulation and changes in consumption behavior.

## References

[1] World Health Organization. Factsheet on obesity and overweight. 2018; https://www.who.int/en/news-room/fact-sheets/detail/obesity-and-overweight.

[2] World Health Organization. Factsheet on diabetes. 2018; https://www.who.int/news-room/fact-sheets/detail/diabetes.

[3] Reedy J, Krebs-Smith SM (2010). Dietary sources of energy, solid fats, and added sugars among children and adolescents in the United States. J Am Diet Assoc.

[4] World Health Organization. Fiscal policies for diet and prevention of noncommunicable diseases: technical meeting report, 5–6 May 2015, Geneva, Switzerland. 2016.

[5] Malik VS, Hu FB (2011). Sugar-sweetened beverages and health: where does the evidence stand?. Am J Clin Nutr.

[6] Malik VS, Pan A, Willett WC, Hu FB (2013). Sugar-sweetened beverages and weight gain in children and adults: a systematic review and meta-analysis. Am J Clin Nutr.

[7] Finkelstein EA, daCosta DiBonaventura M, Burgess SM, Hale BC (2010). The costs of obesity in the workplace. J Occup Environ Med.

[8] Cawley J, Meyerhoefer C (2012). The medical care costs of obesity: an instrumental variables approach. J Health Econ..

[9] Global Food Research Program UNC. Sugary drink taxes around the world. http://globalfoodresearchprogram.web.unc.edu/files/2018/11/SugaryDrink_tax_maps_Nov2018_global.pdf. Accessed 15 Nov 2018.

[10] Colchero MA, Popkin BM, Rivera JA, Ng SW (2016). Beverage purchases from stores in Mexico under the excise tax on sugar sweetened beverages: observational study. BMJ..

[11] Colchero MA, Rivera-Dommarco J, Popkin BM, Ng SW (2017). In Mexico, evidence of sustained consumer response two years after implementing a sugar-sweetened beverage tax. Health Aff.

[12] Alvarado M, Unwin N, Sharp SJ (2019). Assessing the impact of the Barbados sugar-sweetened beverage tax on beverage sales: an observational study. Int J Behav Nutr Phys Act..

[13] Silver LD, Ng SW, Ryan-Ibarra S (2017). Changes in prices, sales, consumer spending, and beverage consumption one year after a tax on sugar-sweetened beverages in Berkeley, California, US: a before-and-after study. PLoS Med.

[14] Falbe J, Thompson HR, Becker CM, Rojas N, McCulloch CE, Madsen KA (2016). Impact of the Berkeley excise tax on sugar-sweetened beverage consumption. Am J Public Health.

[15] Zhong Y, Auchincloss AH, Lee BK, Kanter GP (2018). The short-term impacts of the Philadelphia beverage tax on beverage consumption. Am J Prev Med.

[16] Roberto CA, Lawman HG, LeVasseur MT (2019). Association of a beverage tax on sugar-sweetened and artificially sweetened beverages with changes in beverage prices and sales at chain retailers in a large urban setting. J Am Med Assoc.

[17] Cawley J, Frisvold D, Hill A, Jones D (2019). The impact of the Philadelphia beverage tax on purchases and consumption by adults and children. J Health Econ.

[18] Caro JC, Corvalán C, Reyes M, Silva A, Popkin B, Taillie LS (2018). Chile’s 2014 sugar-sweetened beverage tax and changes in prices and purchases of sugar-sweetened beverages: an observational study in an urban environment. PLoS Med.

[19] Nakamura R, Mirelman A, Cuadrado C, Silva N, Dunstan J, Suhrcke ME (2018). Evaluating the 2014 sugar-sweetened beverage tax in Chile: an observational study in urban areas. PLoS Med.

[20] Public Health England (2018). Sugar reduction and wider reformulation programme: report on progress towards the first 5% reduction and next steps.

[21] Stacey N, Mudara C, Ng SW, van Walbeek C, Hofman K, Edoka I (2019). Sugar-based beverage taxes and beverage prices: evidence from South Africa’s Health Promotion Levy. Soc Sci Med.

[22] Rudd Center for Food Policy & Obesity. Revenue calculator for sugar-sweetened beverage taxes. http://www.uconnruddcenter.org/revenue-calculator-for-sugar-sweetened-beverage-taxes. Accessed 5 Oct 2018.

[23] Staff Beverage Digest Editorial (2017). Beverage digest Fact Book: statistical yearbook of non-alcoholic beverages, 2016.

[24] United States Census Bureau. 2016 American community survey 1-year estimates. 2017. Accessed 30 Nov 2018.

[25] National Center for Health Statistics. National Health and Nutrition Examination Survey. Questionnaires, datasets and related documentation: NHANES 2013-14. https://wwwn.cdc.gov/nchs/nhanes/continuousnhanes/default.aspx?BeginYear=2013. Accessed 30 Nov 18.

[26] Bureau of Labor Statistics. Consumer Price Index for All Urban Consumers (CPI-U): U. S. city average, by detailed expenditure category—seasonally-adjusted estimates: carbonated beverages and juices and non-carbonated beverages. In. 10/11/18 ed: Bureau of Labor Statistics; 2018.

[27] Powell LM, Chriqui JF, Khan T, Wada R, Chaloupka FJ (2013). Assessing the potential effectiveness of food and beverage taxes and subsidies for improving public health: a systematic review of prices, demand and body weight outcomes. Obes Rev.

[28] Cawley J, Thow AM, Wen K, Frisvold D (2019). The economics of taxes on sugar-sweetened beverages: a review of the effects on prices, sales, cross-border shopping, and consumption. Annu Rev Nutr.

